# Facile Synthesis and Enhanced Visible-Light Photocatalytic Activity of Novel p-Ag_3_PO_4_/n-BiFeO_3_ Heterojunction Composites for Dye Degradation

**DOI:** 10.1186/s11671-018-2671-6

**Published:** 2018-08-29

**Authors:** Lijing Di, Hua Yang, Tao Xian, Xiujuan Chen

**Affiliations:** 1 State Key Laboratory of Advanced Processing and Recycling of Non-ferrous Metals, Lanzhou University of Technology, Lanzhou, 730050 China; 2 College of Physics and Electronic Information Engineering, Qinghai Normal University, Xining, 810008 China

**Keywords:** BiFeO_3_, Ag_3_PO_4_, Heterojunction, Photocatalysis

## Abstract

In this work, Ag_3_PO_4_ microparticles were decorated onto the surface of BiFeO_3_ microcuboids through a precipitation method to obtain p-Ag_3_PO_4_/n-BiFeO_3_ heterojunction composites. The composites were employed for the degradation of acid orange 7 (AO7) under visible-light irradiation. It is found that the composites exhibit much higher photocatalytic efficiency than bare BiFeO_3_. Meanwhile, the intrinsical visible-light-driven photocatalytic activity of Ag_3_PO_4_/BiFeO_3_ composites was further confirmed by the degradation of phenol. In addition, the photo-Fenton-like catalysis property of the composite was also evaluated. The photocurrent analysis indicates that the combination of BiFeO_3_ with Ag_3_PO_4_ leads to the inhibition of recombination of photoinduced electrons and holes. The obvious enhancement in the photocatalytic activity of the composite is mainly ascribed to the efficient photogenerated charge separation and interfacial charge migration caused by the formation of Ag_3_PO_4_/BiFeO_3_ p-n heterojunctions.

## PACS

81.05.Hd82.65.+r82.50.–m

## Background

Recently, the semiconductor photocatalysis has received considerable attention as a promising technology for energy conversion and pollution treatment [1–3]. As we know, the widely investigated photocatalyst TiO_2_ is merely active under ultraviolet (UV) light irradiation which only constitutes about 5% of solar light and thus greatly limits its photocatalytic applications under sunlight. Consequently, it is necessary to develop the visible-light-driven photocatalysts [4–8].

Bismuth- or ferrum-based semiconductor oxides generally possess a moderate bandgap energy (~ 2.0 eV) and are regarded as an important class of visible-light-responsive photocatalysts [9–19]. Among them, BiFeO_3_ with a perovskite-type structure is found to exhibit interesting photocatalytic performance for the dye degradation and water splitting under visible-light irradiation [20–25]. However, its photocatalytic activity is not impressive due to the high recombination rate of photogenerated electrons (e^−^) and holes (h^+^). Fortunately, it is demonstrated that coupling of BiFeO_3_ with a narrow-bandgap semiconductor of matched band edge potentials to form a heterojunction is one of the most promising strategies to promote the separation of photogenerated charges, thus leading to improved photocatalytic activity of BiFeO_3_ [26–31]. For example, Chaiwichian et al. reported that BiFeO_3_-Bi_2_WO_6_ nanocomposites exhibited enhanced activity in dye degradation [29]. Wang et al. observed that AgCl/Ag/BiFeO_3_ showed much higher visible-light photocatalytic activity than bare BiFeO_3_ [30]. Fan and co-workers found that the combination of g-C_3_N_4_ with BiFeO_3_ can obviously improve the catalytic activity compared with pure BiFeO_3_ [31].

Silver orthophosphate (Ag_3_PO_4_), as an excellent visible-light-driven photocatalyst, has attracted considerable attention in the photocatalytic field [32–37]. It has been shown that Ag_3_PO_4_ can achieve extremely high quantum yield (~ 90%) for oxygen generation from water splitting [32, 33]. Furthermore, it possesses superior photooxidation capability for organic pollution degradation due to its highly positive valence band position [34]. In most cases, owing to its appropriate energy band position and narrow bandgap, Ag_3_PO_4_ is widely employed as the cocatalyst to combine with other photocatalysts to form composites, leading to an obvious improvement of photocatalytic behavior, such as Ag_3_PO_4_/Bi_2_WO_6_, Ag_3_PO_4_/BiPO_4_, Ag_3_PO_4_/Bi_2_O_2_CO_3_, Ag_3_PO_4_/g-C_3_N_4_, Ag_3_PO_4_/BiVO_4_, Bi_4_Ti_3_O_12_/Ag_3_PO_4_, Ag_3_PO_4_/ZnFe_2_O_4_, Ag_3_PO_4_/WO_3_, Ag_3_PO_4_/ZnO, and Bi_2_MoO_6_/Ag_3_PO_4_ [38–47]. It is reported that BiFeO_3_ is an n-type semiconductor and Ag_3_PO_4_ is known as a p-type semiconductor [43, 48]. The construction of Ag_3_PO_4_/BiFeO_3_ p-n heterojunction composites may be a feasible method to obtain efficient photocatalyst. However, to the best of our knowledge, little work has been devoted to the investigation of photocatalytic performance of Ag_3_PO_4_/BiFeO_3_ composites.

In this work, Ag_3_PO_4_/BiFeO_3_ p-n heterojunction composites were facilely prepared via the precipitation of Ag_3_PO_4_ microparticles on the BiFeO_3_ microcuboids. Acid orange 7 (AO7) and phenol were selected as the model pollutant to evaluate the photocatalytic activity of the composites under visible-light irradiation. Moreover, the photo-Fenton-like catalysis activity of the composite was also investigated. The underlying mechanism of the composites for the degradation of organic pollutants was discussed.

## Methods

### Preparation of Ag_3_PO_4_/BiFeO_3_ Composites

BiFeO_3_ microcuboids were synthesized via a hydrothermal route. 0.005 mol of Bi(NO_3_)_3_•5H_2_O and 0.005 mol of Fe(NO_3_)_3_•9H_2_O were dissolved in 20 mL of dilute nitric acid solution (5 mL HNO_3_ + 15 mL deionized water). Sixty milliliters of KOH solution with concentration of 4.5 mol/L was added to the above solution drop by drop under magnetic stirring. After 8 min of ultrasonic treatment and another 30 min of vigorous magnetic stirring, the mixture solution was sealed in a Teflon-lined stainless steel autoclave of 100 mL capacity and submitted to hydrothermal reaction at 200 °C for 6 h. After the autoclave was cooled naturally to room temperature, the precipitate was collected by centrifugation, washed with deionized water (two times) and absolute ethanol (three times), and then dried at 80 °C for 12 h to obtain final BiFeO_3_ product. Ag_3_PO_4_ microparticles were prepared by a precipitation method. Three millimoles of AgNO_3_ was dissolved into 30 mL deionized water, and 1 mmol Na_3_PO_4_·12H_2_O was added into 30 mL deionized water with the aid of magnetic stirring. After the solution was homogeneous, the latter solution was added dropwise into the former under vigorous magnetic stirring for 7 h. During the reaction, the color of the solution changed into yellow. Finally, the mixture was centrifuged to collect the precipitate. The obtained precipitate was washed several times with deionized water and then dried in a vacuum oven at 60 °C for 8 h.

Ag_3_PO_4_/BiFeO_3_ composites were synthesized as follows: 0.1 g of BiFeO_3_ microcuboids were dispersed in 30 mL deionized water and then ultrasonicated for 2 h. After that, a certain amount of AgNO_3_ was dissolved into the above suspension. To this mixture was added drop by drop a certain concentration of Na_3_PO_4_ solution (30 mL) under vigorous magnetic stirring for 7 h. The as-obtained composites were separated by centrifugation, washed repeatedly with deionized water, and dried in a vacuum oven at 60 °C for 8 h. To investigate the effect of Ag_3_PO_4_ content on the photocatalytic property of obtained composites, a series of sample was fabricated with different Ag_3_PO_4_ mass ratios of 5%, 10%, 20%, and 40% and the corresponding samples were termed as 5wt%Ag_3_PO_4_/BiFeO_3_, 10wt%Ag_3_PO_4_/BiFeO_3_, 20wt%Ag_3_PO_4_/BiFeO_3_, and 40wt%Ag_3_PO_4_/BiFeO_3_, respectively. For comparison, the composite termed as 20wt%Ag_3_PO_4_/BiFeO_3_-M was also prepared by direct mechanical mixing of BiFeO_3_ microcuboids and Ag_3_PO_4_ microparticles, where Ag_3_PO_4_ occupies a mass fraction of 20% in the composite.

### Photoelectrochemical Measurements

The photocurrent test was carried out on the electrochemical workstation (CST 350) with a three-electrode cell as described in the literature [49]. In this three-electrode system, a platinum foil and a standard calomel electrode were used as the counter electrode and reference electrode, respectively. The working electrode was fabricated as follows: 15 mg photocatalysts, 0.75 mg carbon black, and 0.75 mg polyvinylidene fluoride (PVDF) were added into 1-methyl-2-pyrrolidione (NMP) to produce slurry, which was then uniformly coated on a 1.0 × 1.0 cm^2^ fluoride-doped tin oxide glass electrode. After that, the electrode was dried at 60 °C for 5 h. A 300-W Xe lamp with a 420-nm cut-off filter was employed as the visible light source. The photoelectrochemical measurement was performed in the 0.1-M Na_2_SO_4_ electrolyte solution, and its pH value was measured to be ~ 5.3.The photocurrent-time (I-t) curves were measured at a fixed bias potential of 0.2 V. The electrochemical impedance spectroscopy (EIS) test was performed by using the sinusoidal voltage pulse with amplitude of 5 mV and in the frequency range from 10^−2^ to 10^5^ Hz.

### Photocatalytic Activity Test

The photocatalytic activity of samples was evaluated toward the degradation of AO7 and phenol under visible-light irradiation. Typically, the initial AO7 or phenol concentration was 5 mg/L with a catalyst loading of 0.5 g/L. The pH values of AO7 and phenol solution were measured to be ~ 6.8 and ~ 6.2, respectively. Prior to illumination, the mixture was stirred in the dark for 0.5 h to achieve the adsorption-desorption equilibrium of organic molecule on the surface of catalysts. This reaction solution was then exposed to a 300-W xenon lamp with a 420-nm cut-off filter, and the corresponding light intensity was measured to be ~ 50 mW cm^−2^. During the photocatalytic experiment, a small amount of reaction solution was collected at the given time intervals and then centrifuged to separate catalysts. The concentration of AO7 or phenol was determined by detecting the absorbance of the supernatant at a given wavelength (λ _AO7_ = 484 nm and λ _phenol_ = 270 nm) using a UV-visible spectrophotometer. To evaluate the photocatalytic reusability of the photocatalysts, the recycling experiment for the degradation of AO7 was performed. After the first photocatalytic test was completed, the photocatalysts were collected by centrifugation, washed with distilled water, and dried. The collected photocatalysts were added into the fresh dye solution for the next cycle of the photocatalytic experiment. To investigate the photo-Fenton-like catalysis ability of the photocatalysts, H_2_O_2_ (5 mmol/L) was added into the reaction solution. The photo-Fenton-like experiment procedure was similar to the above photocatalytic process.

### Characterization

The phase purity of the samples was investigated by X-ray diffractometer (XRD, Bruker D8 Advanced) using Cu Kα radiation. The morphology of the samples was observed by a field-emission scanning electron microscope (SEM, JEOL JSM-6701F) and field-emission transmission electron microscope (TEM, JEOL JEM-2010). The composition of the samples was measured by energy dispersive X-ray spectroscopy. The chemical state of the element was tested using X-ray photoelectron spectroscopy (XPS, PHI-5702), where the binding energy scale of the XPS data was calibrated against the adventitious C 1s peak at the binding energy of 284.8 eV. The ultraviolet-visible (UV-vis) diffuse reflectance spectra of the products were obtained using a UV-vis spectrophotometer (PERSEE TU-1901) with BaSO_4_ as a reference. The PL spectra of the samples were recorded on a fluorescence spectrophotometer (SHIMADZU RF-6000) with the excitation wavelength of ~ 350 nm.

## Results and Discussion

### XRD Analysis

**Fig. 1:**
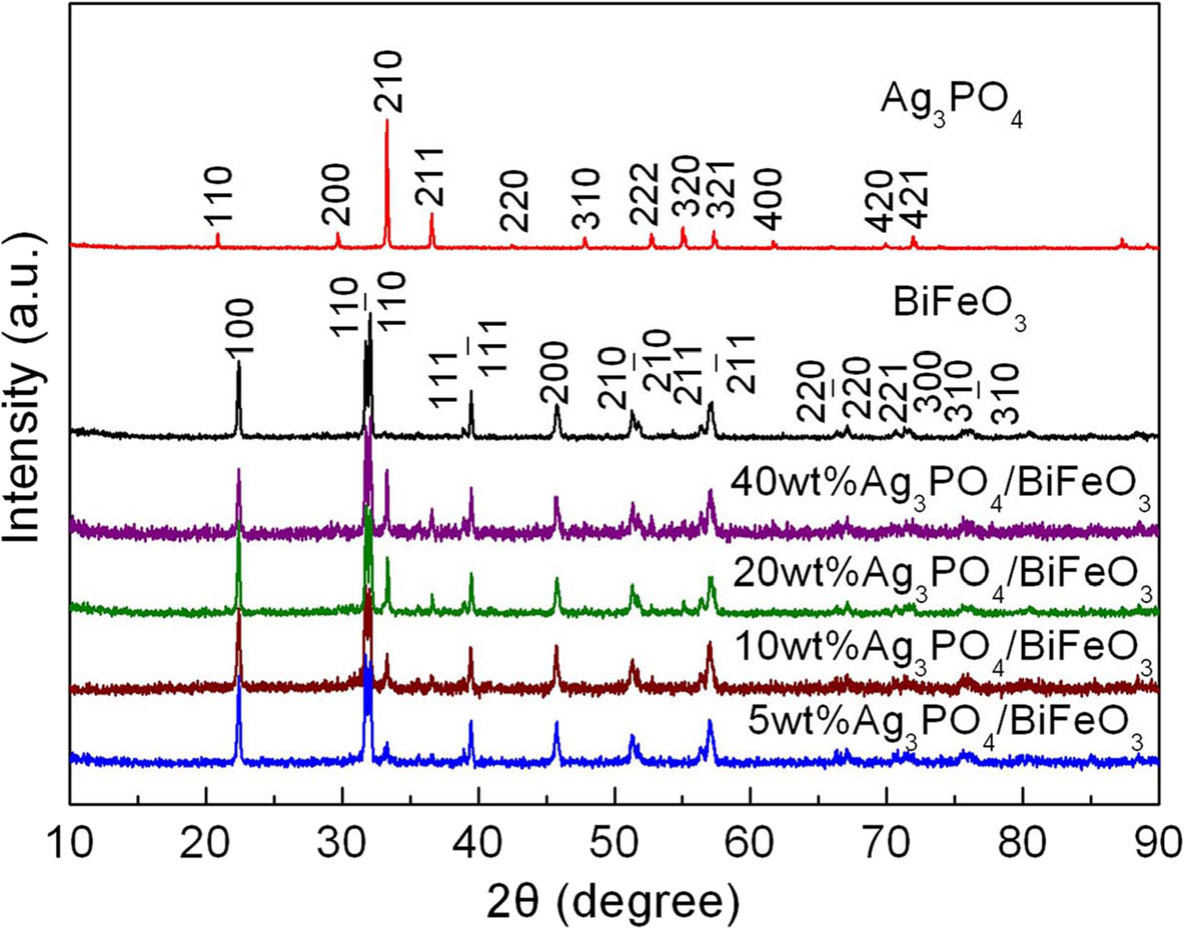
XRD patterns of BiFeO_3_, Ag_3_PO_4_, and Ag_3_PO_4_/BiFeO_3_ composites

Figure 1 presents the XRD patterns of BiFeO_3_, Ag_3_PO_4_, and Ag_3_PO_4_/BiFeO_3_ composites with different Ag_3_PO_4_ contents. For bare BiFeO_3_ sample, all the diffraction peaks match well with the rhombohedral structure of BiFeO_3_ (PDF card no. 74-2016), and for bare Ag_3_PO_4_ sample, the diffraction peaks can be perfectly indexed to cubic Ag_3_PO_4_ phase (PDF card no. 06-0505); this indicates that high-purity BiFeO_3_ and Ag_3_PO_4_ have been successfully prepared. In the case of the composites, the XRD patterns can be assigned to the characteristic diffraction peaks of BiFeO_3_ and Ag_3_PO_4_, and no diffraction peaks of impurity appear in the patterns. Moreover, it is seen that by increasing the content of Ag_3_PO_4_, the intensity of the characteristic peaks of Ag_3_PO_4_ increases gradually. The results suggest that the composites consist of rhombohedral BiFeO_3_ and cubic Ag_3_PO_4_, and no other phase is generated during the preparation of the composites.

### Morphology Observation

**Fig. 2:**
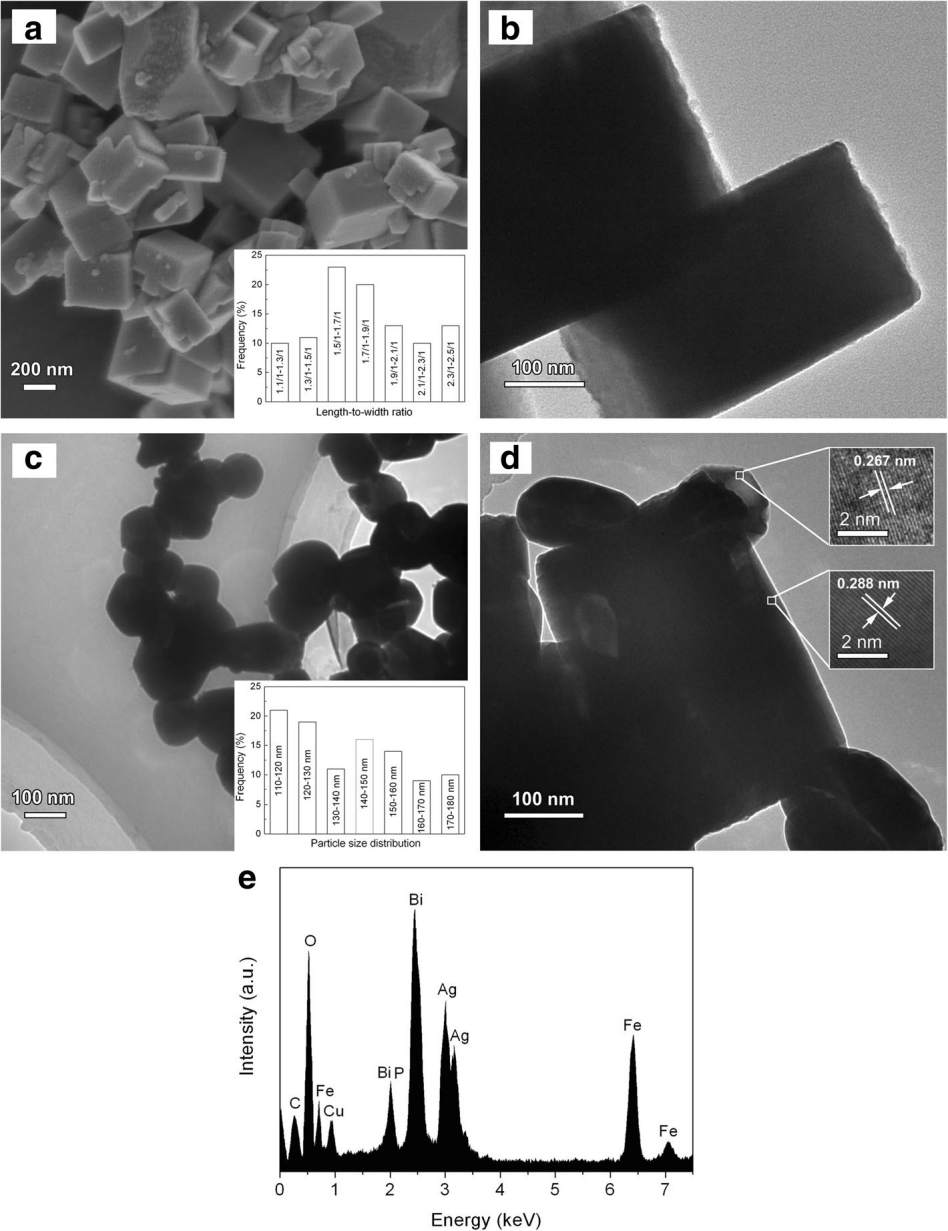
**a** SEM (the inset is the length-to-width ratio distribution of BiFeO_3_ microcuboids) and **b** TEM image of BiFeO_3_ microcuboids. TEM image of **c** Ag_3_PO_4_ microparticles (the inset is the size distribution of Ag_3_PO_4_ microparticles) and **d** 20wt%Ag_3_PO_4_/BiFeO_3_ sample; inset shows its HRTEM image. **e** EDX spectrum of 20wt%Ag_3_PO_4_/BiFeO_3_ sample

The morphology of the samples was observed by SEM and TEM. Figure 2a, b shows the SEM image and TEM image of bare BiFeO_3_, revealing that the prepared BiFeO_3_ particles exhibit cuboid-like shape with 200–500 nm in size and have a smooth surface. The inset of Fig. 2a displays the length-to-width ratio distribution of BiFeO_3_ particles, which reveals that the length-to-width ratio ranges from 1.1/1 to 2.5/1. As can be seen from the TEM image in Fig. 2c, bare Ag_3_PO_4_ consists of irregular sphere-like particles. The size distribution of Ag_3_PO_4_ particles is shown in the inset of Fig. 2c, indicating a wide distribution of particle size ranging from 110 to 180 nm. From the TEM image of the 20wt%Ag_3_PO_4_/BiFeO_3_ composite (Fig. 2d), one can see that the irregular microspheres are attached to the cuboid-shaped particle. The high-resolution TEM (HRTEM) images obtained from the different particles indicate two distinct sets of lattice fringes (insets in Fig. 2d). The interplanar spacing of ~ 0.288 nm matches the BiFeO_3_ (110) planes, whereas the interplanar distance of ~ 0.267 nm corresponds to the Ag_3_PO_4_ (210) planes. In addition, the EDX analysis suggests that the composite includes all the elements of Ag_3_PO_4_ and BiFeO_3_ phases (Fig. 2e). The observed C and Cu signals in the EDX spectrum of Fig. 2e could arise from the microgrid used for supporting the sample [50]. These results reveal that Ag_3_PO_4_ particles are decorated on the surface of BiFeO_3_ microcuboids, resulting in the formation of Ag_3_PO_4_/BiFeO_3_ p-n heterostructures.

### XPS Analysis

**Fig. 3:**
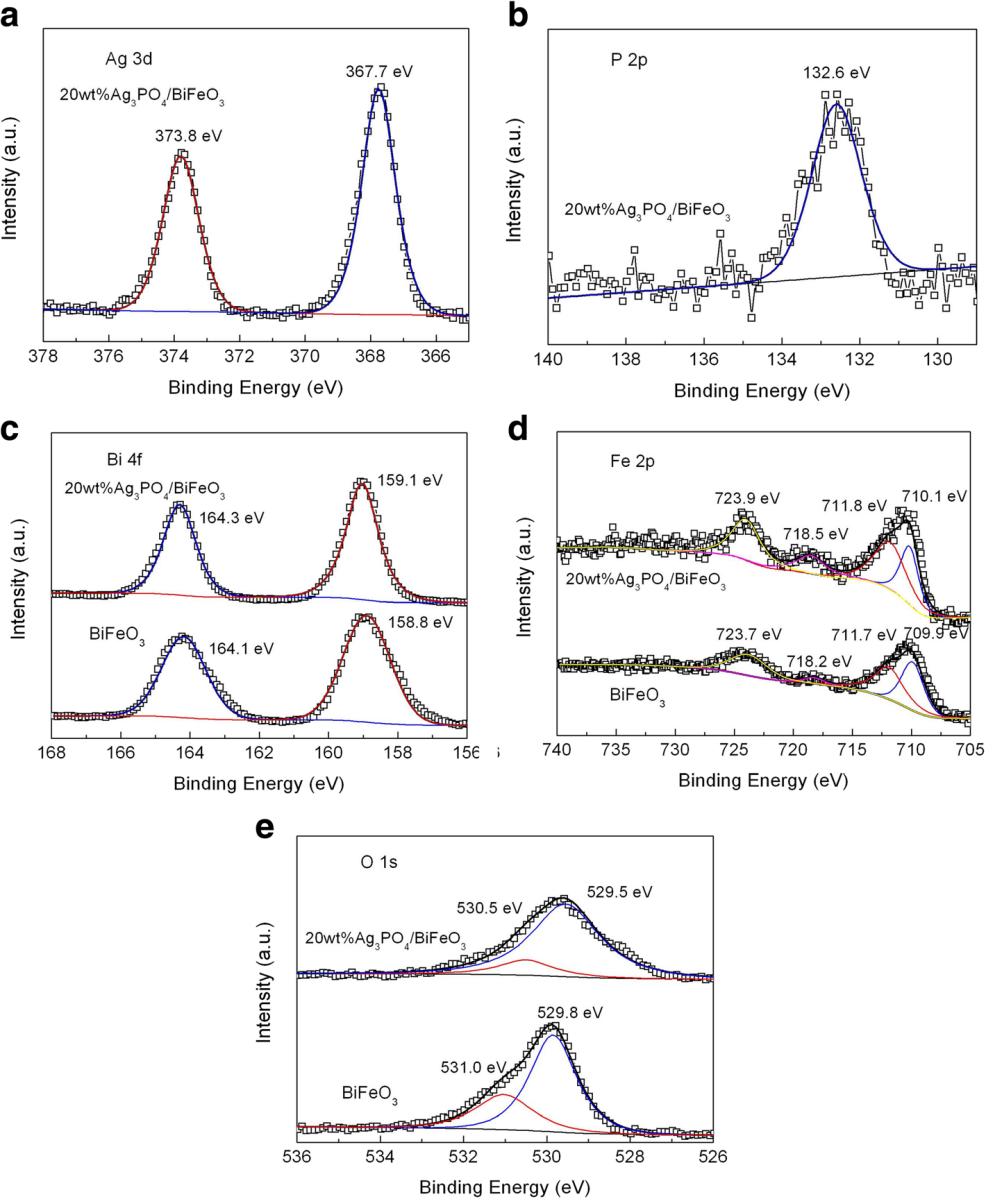
High-resolution XPS spectra of BiFeO_3_ and 20wt%Ag_3_PO_4_/BiFeO_3_ sample. **a** Ag 3d and **b** P 2p of 20wt%Ag_3_PO_4_/BiFeO_3_ sample. **c** Bi 4f, **d** Fe 2p, and **e** O1s of BiFeO_3_ and 20wt%Ag_3_PO_4_/BiFeO_3_ sample

The XPS analysis was performed to reveal the chemical states of BiFeO_3_ and 20wt%Ag_3_PO_4_/BiFeO_3_, as shown in Fig. 3. Figure 3a shows the high-resolution XPS spectrum of Ag 3d in the composite. The two obvious peaks at 373.8 and 367.7 eV are attributed to the Ag 3d_3/2_ and Ag 3d_5/2_ binding energies of Ag^+^. Figure 3b presents the P 2p high-resolution XPS spectrum of the composite. The peak at around 133.2 eV corresponds to the characteristic binding energy of P^5+^ oxidation state in Ag_3_PO_4_ [51]. Figure 3c, d, shows the Bi 4f and Fe 2p high-resolution XPS spectra, respectively. For bare BiFeO_3_, the Bi 4f spectrum shows two strong peaks at binding energies of 164.1 eV and 158.8 eV, belonging to the Bi 4f_5/2_ and Bi 4f_7/2_, respectively, which indicates that Bi ion possesses the oxidation state of + 3. For Fe 2p spectrum, the peak located at 723.7 eV is assigned to the Fe 2p_1/2_ of Fe^3+^. Another strong XPS signal at ~ 711.6 eV can be fitted into two peaks at 711.7 and 709.9 eV. The peak at 709.9 eV corresponds to the binding energy of Fe 2p_3/2_ of Fe^2+^. The binding energy at 711.7 eV belongs to the Fe 2p_3/2_ of Fe^3+^. In addition, a satellite peak is found at around 718.2 eV, which is attributed to the mixed oxidation states of Fe. From the XPS analysis of the Fe element, it can be seen that Fe exists in the form of Fe^3+^ and Fe^2+^ in bare BiFeO_3_. It is worth noting that the Bi 4f and Fe 2p binding energies in 20wt%Ag_3_PO_4_/BiFeO_3_ exhibit a slight shift in comparison to bare BiFeO_3_, which is mainly attributed to the interaction between BiFeO_3_ and Ag_3_PO_4_. Figure 3e displays the O 1s high-resolution XPS spectra of BiFeO_3_ and 20wt%Ag_3_PO_4_/BiFeO_3_. For bare BiFeO_3_, the O 1s signal can be divided into two peaks at 529.8 and 531.0 eV. The binding energy of 529.8 eV corresponds to the lattice oxygen while the small peak at higher binding energy of 531.0 eV is caused by surface defects and chemisorbed oxygen species. Compared with bare BiFeO_3_, the O 1s peak in the composite experiences a shift, which is also due to the interaction between Ag_3_PO_4_ and BiFeO_3_.

### Optical Absorption Property

**Fig. 4:**
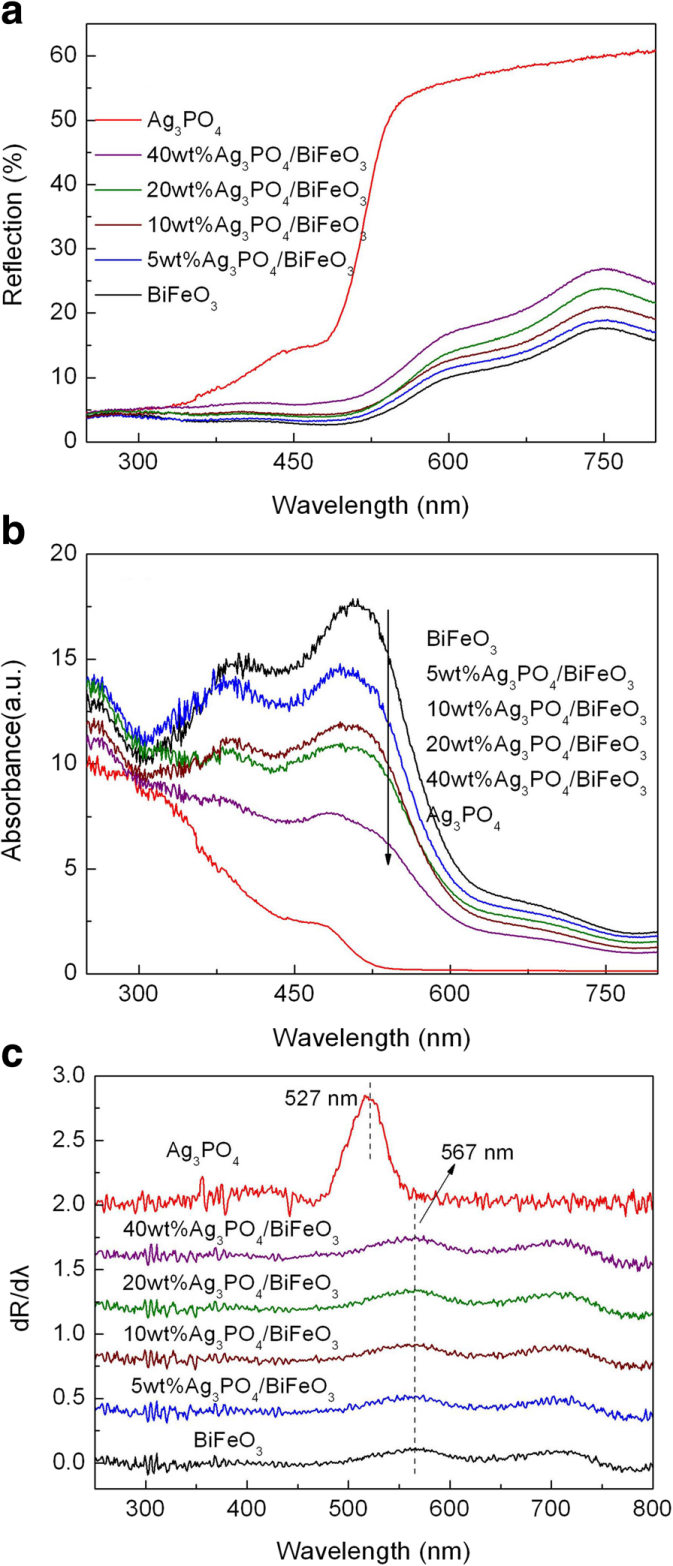
**a** UV-visible diffuse reflectance spectra of BiFeO_3_, Ag_3_PO_4_, and Ag_3_PO_4_/BiFeO_3_ composites. **b** The corresponding absorption spectra and **c** the corresponding first derivative of the diffuse reflectance spectra

The optical absorption behavior of the samples was investigated by measuring their UV-vis diffuse reflectance spectra, as presented in Fig. 4a. The corresponding absorption spectra transformed from the diffuse reflectance spectra according to the Kubelka-Munk (K-M) theory is shown in Fig. 4b [52]. It is seen that all the samples exhibit an important light absorption at λ < 600 nm. In order to obtain the absorption edge of the samples, the first derivative of the reflectance (R) with respect to wavelength λ (i.e., dR/dλ) was carried out, as shown in Fig. 4c. The absorption edge can be determined from the peak wavelength in the derivative spectra [53]. It can be seen that the light absorption edge of bare Ag_3_PO_4_ is located at ~ 527 nm, corresponding to the bandgap energy (*E*_g_) of ~ 2.35 eV. Bare BiFeO_3_ exhibits an absorption edge at around 567 nm, corresponding to the *E*_g_ of ~ 2.18 eV. In addition to the absorption edge, a weak peak at ~ 700 nm is observed, which is probably attributed to the existence of surface states in the middle of the bandgap of BiFeO_3_. When coupled with Ag_3_PO_4_, the absorption edge of BiFeO_3_ does not undergo obvious change, which indicates that the introduction of Ag_3_PO_4_ has no apparent effect on the bandgap structure of BiFeO_3_.

### Photocatalytic Activity Measurement

**Fig. 5:**
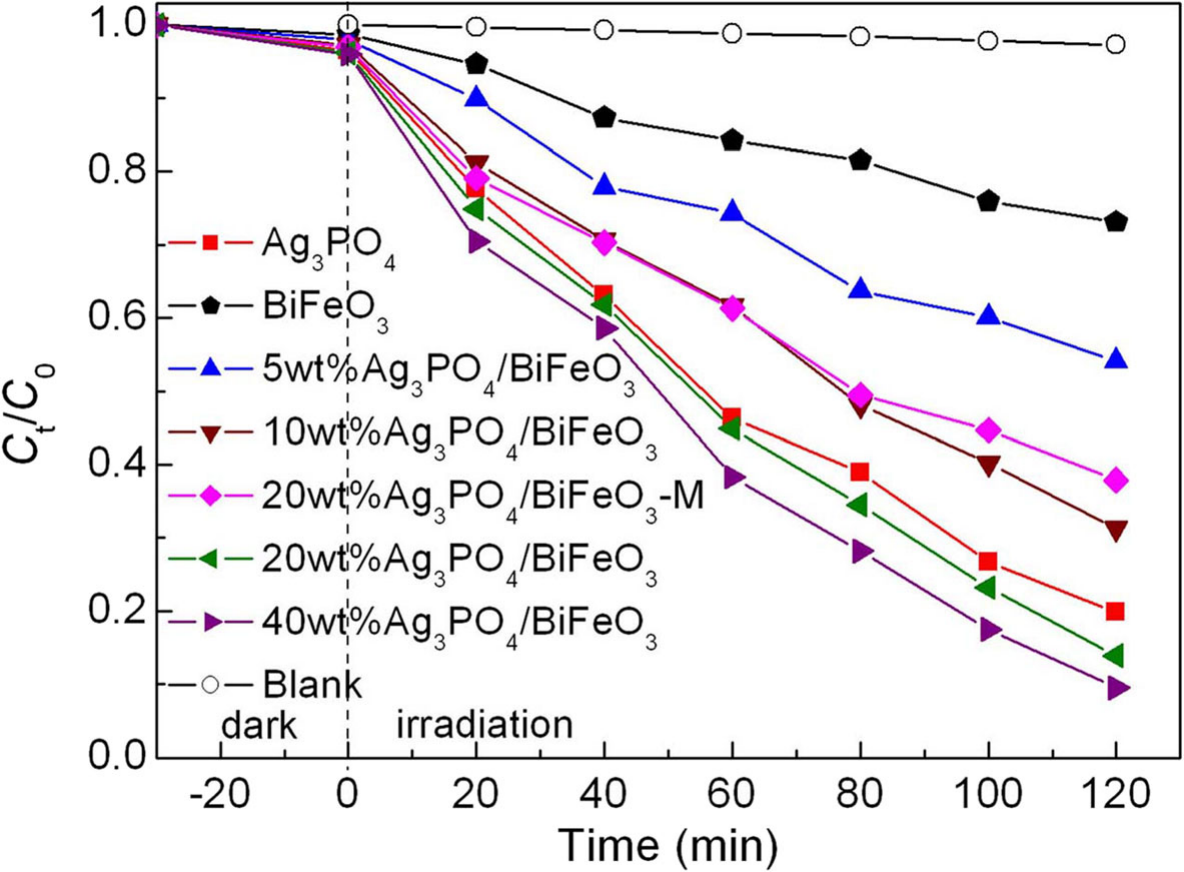
Photocatalytic activities of BiFeO_3_ and Ag_3_PO_4_/BiFeO_3_ composites toward the degradation of AO7 under visible-light irradiation, along with the blank and absorption experiment results

AO7 was selected as a target pollutant for evaluating the photocatalytic performance of the samples. The photocatalytic degradation of AO7 was investigated under visible-light irradiation, and the result is shown in Fig. 5. Prior to photocatalytic reaction, blank and absorption experiments were performed. It is seen that no obvious degradation of dye is detected under irradiation without the catalysts or in the presence of catalysts without irradiation, suggesting that self-degradation and absorption of AO7 during the photocatalytic process are negligible. Bare BiFeO_3_ has weak photocatalytic activity, and only ~ 27% of AO7 is degraded with 120 min of irradiation. When BiFeO_3_ microcuboids are combined with Ag_3_PO_4_ microparticles, the formed Ag_3_PO_4_/BiFeO_3_ composites exhibit superior photocatalytic activity to bare BiFeO_3_. After 120 min of exposure, the degradation percentage of AO7 over the samples is in the order 40wt%Ag_3_PO_4_/BiFeO_3_ (~ 91%) > 20wt%Ag_3_PO_4_/BiFeO_3_ (~ 87%) > 10wt%Ag_3_PO_4_/BiFeO_3_ (~ 69%) > 5wt%Ag_3_PO_4_/BiFeO_3_ (~ 46%) > BiFeO_3_ (~ 27%). It is found that the photocatalytic performance of the composites exhibit an increasing trend with the increase of Ag_3_PO_4_ content. Among these composites, the photocatalytic efficiency of 40wt%Ag_3_PO_4_/BiFeO_3_ is very close to that of 20wt%Ag_3_PO_4_/BiFeO_3_. Thus, in the present study, the most appropriate mass ratio of Ag_3_PO_4_ can be considered as 20% in the composites. Moreover, it is worth noting that the mechanical mixture sample 20wt%Ag_3_PO_4_/BiFeO_3_-M exhibits much lower photocatalytic activity than 20wt%Ag_3_PO_4_/BiFeO_3_. This reveals that the construction of heterojunction between BiFeO_3_ and Ag_3_PO_4_ is necessary for the enhancement of photocatalytic activity. Moreover, compared with BiFeO_3_/a-Fe_2_O_3_ and BiFeO_3_-Bi_2_WO_6_ composites [26, 29], the Ag_3_PO_4_/BiFeO_3_ heterojunction composites prepared in the present study manifest a higher photocatalytic activity toward the dye degradation.

**Fig. 6:**
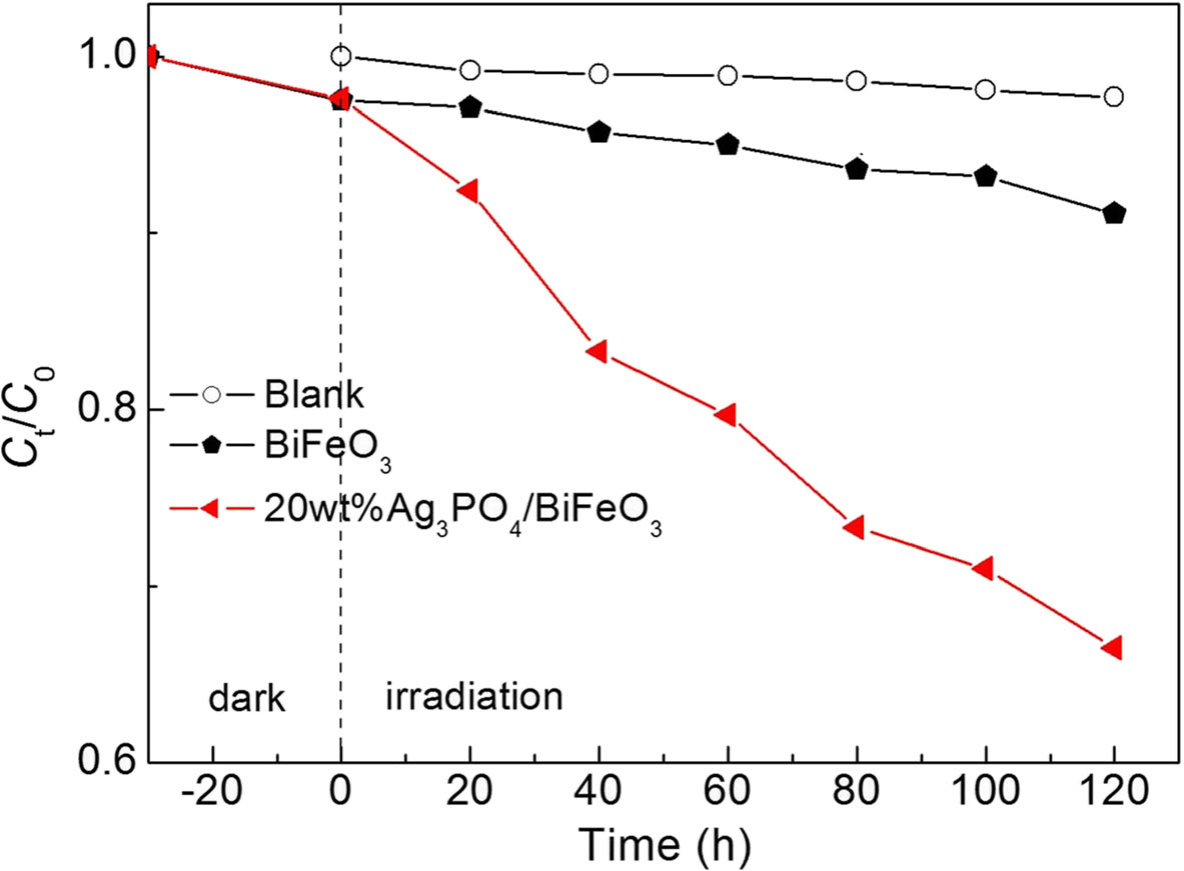
Photocatalytic degradation of phenol over BiFeO_3_ and 20wt%Ag_3_PO_4_/BiFeO_3_ sample under visible-light irradiation, along with the blank and absorption experiment result

To further confirm the photocatalytic property of the composites, the photocatalytic degradation of colorless phenol over 20wt%Ag_3_PO_4_/BiFeO_3_ and BiFeO_3_ under visible-light irradiation was also investigated. As shown in Fig. 6, the self-degradation and absorption of phenol can be neglected based on the results of blank and absorption experiments. It can be seen that just ~ 9% of phenol is degraded catalyzed by BiFeO_3_ after 120 min of exposure. Whereas, when 20wt%Ag_3_PO_4_/BiFeO_3_ is used as the photocatalyst, the degradation percentage of phenol can be obviously enhanced under the same conditions. The result suggests that the degradation of the dye on the visible-light-irradiated Ag_3_PO_4_/BiFeO_3_ composites is attributed to their intrinsical photocatalytic activity instead of dye sensitization.

**Fig. 7:**
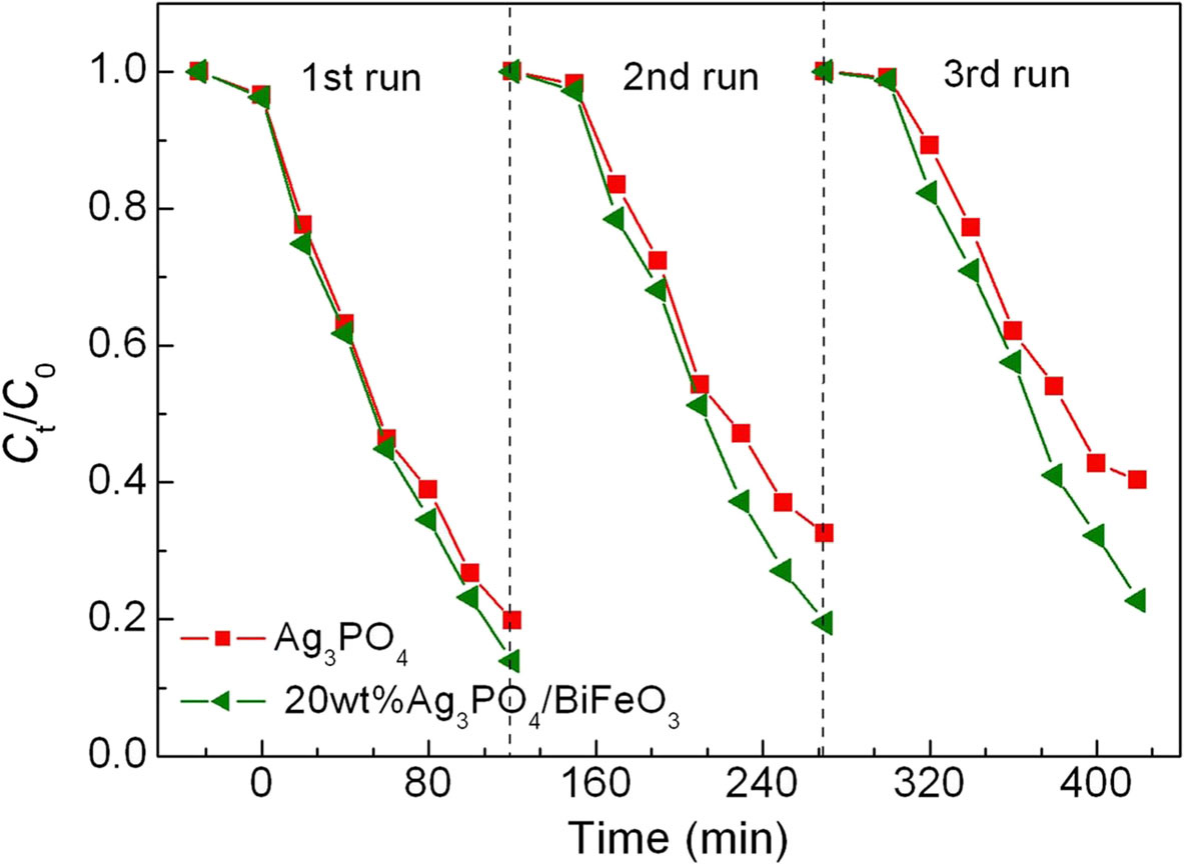
Photocatalytic degradation of AO7 over Ag_3_PO_4_ and 20wt%Ag_3_PO_4_/BiFeO_3_ sample during three cycles

**Fig. 8:**
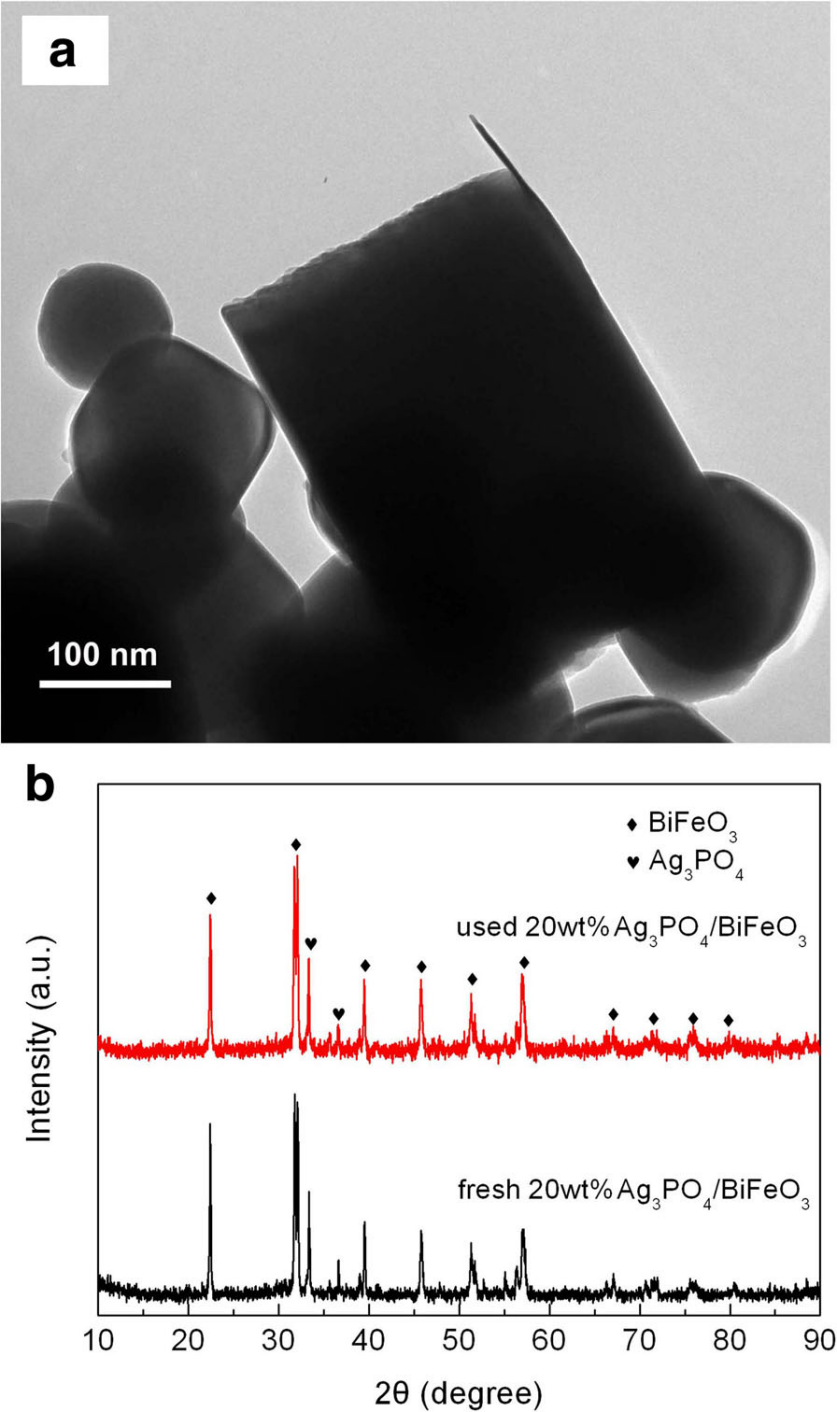
**a** TEM image and **b** XRD pattern of 20wt%Ag_3_PO_4_/BiFeO_3_ sample after cycling photocatalytic experiments

To evaluate the reusability of the photocatalysts, the recycling photocatalytic degradation experiments of AO7 over 20wt%Ag_3_PO_4_/BiFeO_3_ and Ag_3_PO_4_ were carried out under the same photocatalytic conditions. As shown in Fig. 7, after three successive recycling runs, the composite still exhibits relatively high photocatalytic activity, while the degradation efficiency over Ag_3_PO_4_ undergoes an obvious decrease. Figure 8a, b shows the TEM image and XRD pattern of the composite after cycling experiment, respectively. It is clear that Ag_3_PO_4_ microparticles are still assembled on the surface of BiFeO_3_ microcuboids without destruction of the heterostructures, and no obvious crystal structure change is observed. This suggests that Ag_3_PO_4_/BiFeO_3_ p-n heterojunction composites possess good photocatalytic reusability.

### Photo-Fenton-like Catalytic Activity

**Fig. 9:**
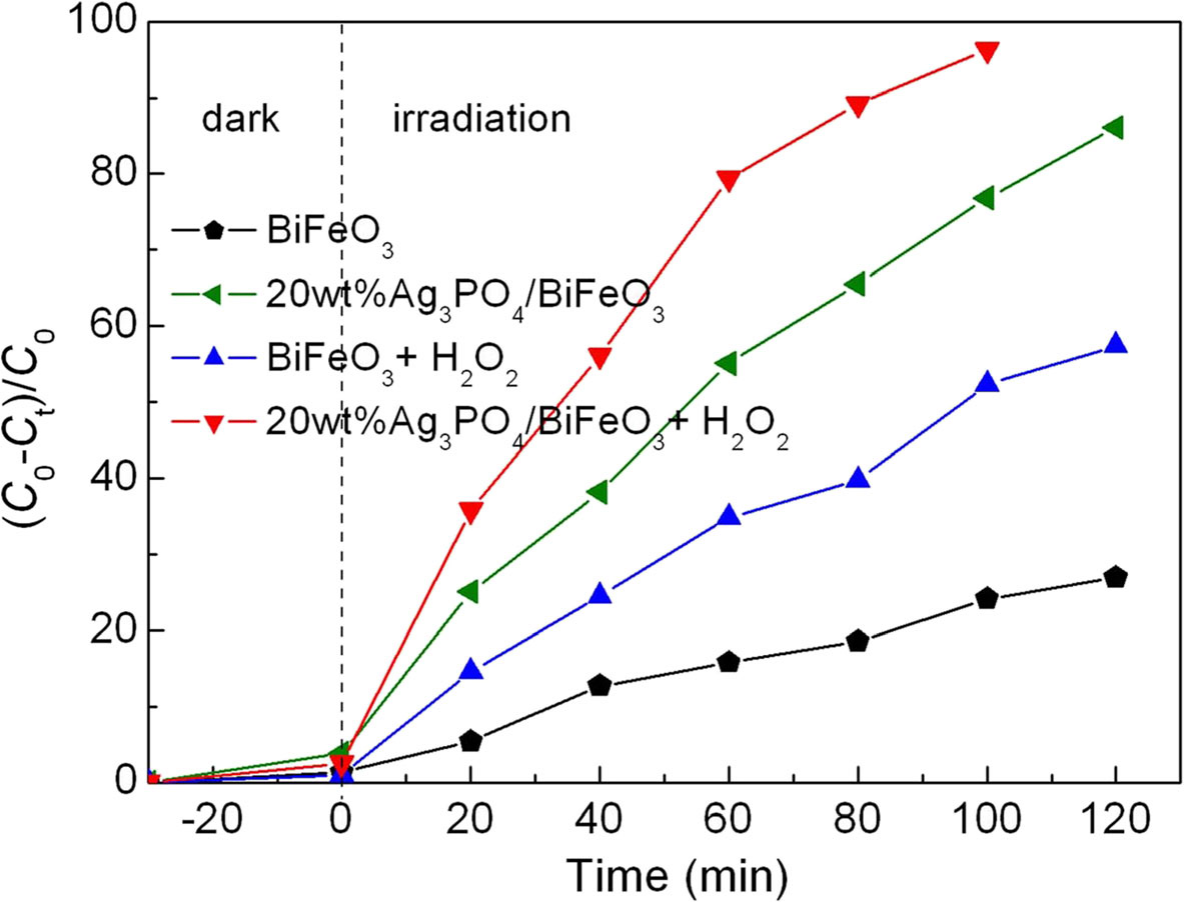
Photocatalytic activities of BiFeO_3_ and 20wt%Ag_3_PO_4_/BiFeO_3_ sample toward the degradation of AO7 under visible-light irradiation in the presence of H_2_O_2_

Apart from its photocatalytic property, BiFeO_3_ also exhibits prominent photo-Fenton-like catalysis ability [54–56]. Figure 9 shows the photo-Fenton-like degradation of AO7 over 20wt%Ag_3_PO_4_/BiFeO_3_ and BiFeO_3_ in the presence of H_2_O_2_. Compared with the reaction systems without H_2_O_2_, the introduction of H_2_O_2_ remarkably enhances the degradation percentage of the dye. This result is mainly due to the photo-Fenton-like reaction mechanism. In the presence of visible-light irradiation and H_2_O_2_, Fe^3+^ on the surface of BiFeO_3_ can be converted to Fe^2+^ with the generation of hydroxyl (•OH) radicals (Eq. 1). Consequently, Fe^2+^ can react with H_2_O_2_ to produce Fe^3+^ and •OH (Eq. 2). During the above cycle reaction, more •OH is produced, which is generally considered to be a primary active species for the dye degradation (as evidenced by active species trapping experiment given in Fig. 11). In the case of bare BiFeO_3_, the high recombination rate of the photogenerated charges limits the yield of photogenerated electrons, which tends to suppress the reduction of Fe^3+^ into Fe^2+^(Eq. 3). This leads to the limited enhancement of degradation percentage. For Ag_3_PO_4_/BiFeO_3_ composites, photogenerated electrons and holes can be efficiently separated, and thus, more photogenerated electrons are available for promoting the quick conversion from Fe^3+^ into Fe^2+^ (Eq. 3) [57]. Benefitting from this electron reduction, the photo-Fenton process for the composites is more efficient than that for bare BiFeO_3_. As a result, Ag_3_PO_4_/BiFeO_3_ p-n heterojunction composites manifest much enhanced photo-Fenton performance.

1 $$ {\mathrm{Fe}}^{3+}+{\mathrm{H}}_2\mathrm{O}+\mathrm{h}\upnu \to {\mathrm{Fe}}^{2+}+\bullet \mathrm{OH}+{\mathrm{H}}^{+} $$

2 $$ {\mathrm{Fe}}^{2+}+{\mathrm{H}}_2{\mathrm{O}}_2\to {\mathrm{Fe}}^{3+}+\bullet \mathrm{OH}+{\mathrm{O}\mathrm{H}}^{-} $$

3 $$ {\mathrm{Fe}}^{3+}+{\mathrm{e}}^{-}\to {\mathrm{Fe}}^{2+} $$

### Photogenerated Charge Performance

**Fig. 10:**
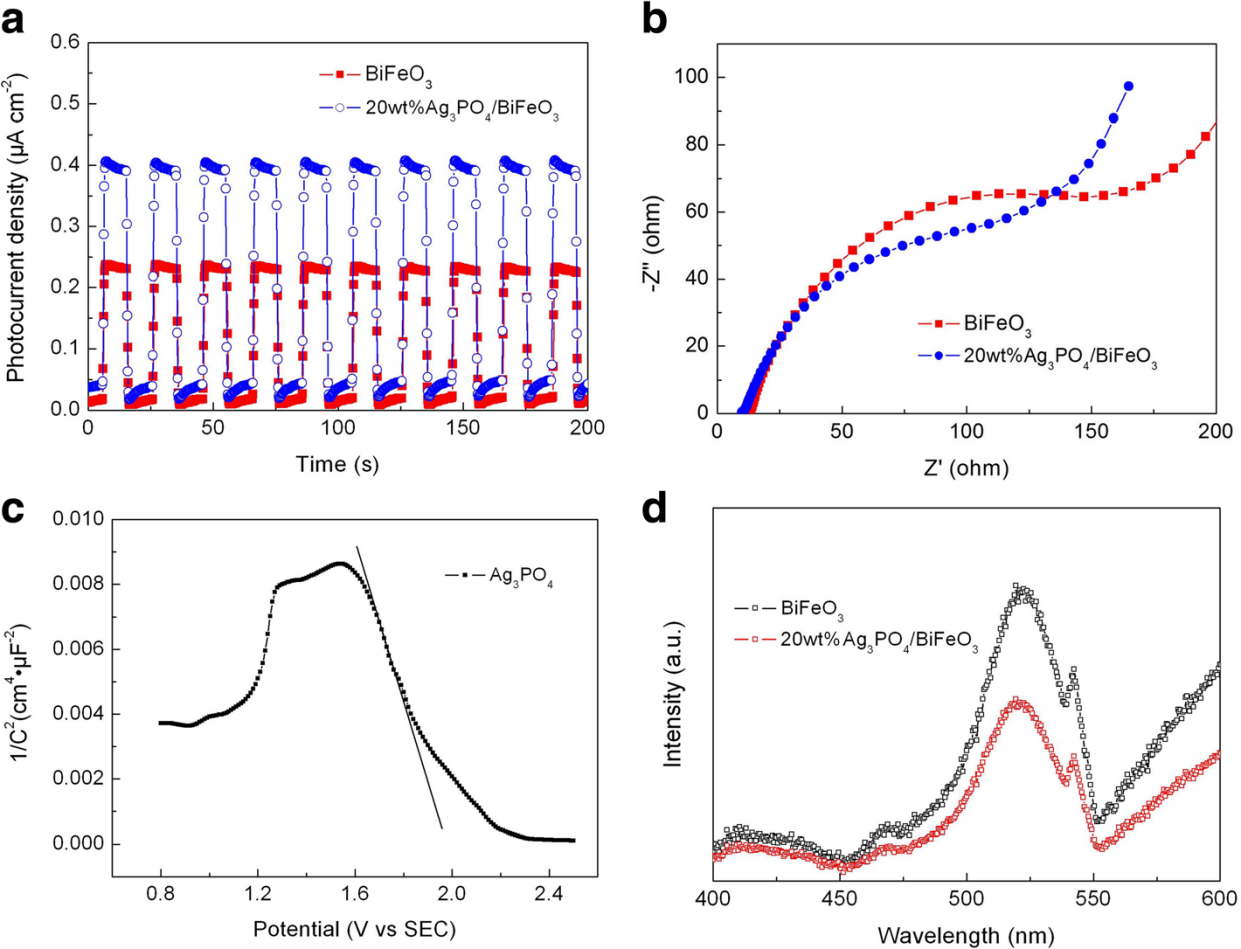
**a** Transient photocurrent response and **b** EIS spectra of BiFeO_3_ and 20wt%Ag_3_PO_4_/BiFeO_3_ sample. **c** Mott-Schottky plot of Ag_3_PO_4_. **d** PL spectra of BiFeO_3_ and 20wt%Ag_3_PO_4_/BiFeO_3_ samples

To evaluate the separation behavior of photogenerated charges of the samples, transient photocurrent responses, ESI spectra, and PL spectra of BiFeO_3_ and 20wt%Ag_3_PO_4_/BiFeO_3_ were measured. Figure 10a shows the photocurrent-time (I-t) curves of the photocatalysts under intermittent visible-light irradiation with several on-off cycles. It can be seen that the photocurrent value of the composite is much higher than that of bare BiFeO_3_, indicating that the construction of Ag_3_PO_4_/BiFeO_3_ p-n heterojunctions is beneficial to inhibit the recombination of photogenerated electrons and holes. Figure 10b presents the ESI spectra of the samples. One can see that the composite exhibits smaller impedance arc radii compared with BiFeO_3_, which suggests the lower charge transfer resistance of the composite. These results reveal that the separation and migration of the photogenerated charges can be improved in the composite, thus providing more photoinduced holes and electrons for the photocatalysis. Figure 10c shows the Mott-Schottky plot at frequency of 3000 Hz for Ag_3_PO_4_. The negative slope of the plot indicates that Ag_3_PO_4_ is a p-type semiconductor, which is consistent with the report [43]. The PL spectra of BiFeO_3_ and 20wt%Ag_3_PO_4_/BiFeO_3_ are shown in Fig. 10d. The two samples exhibit obvious emission peaks at ~ 522 nm, which are mainly attributed to the recombination of the photogenerated electron/hole pairs. It is worth noting that the PL intensity of the composite is much smaller than that of bare BiFeO_3_. This further confirms that the construction of Ag_3_PO_4_/BiFeO_3_ heterojunction promotes the separation of photoinduced charges.

### Active Species Trapping

**Fig. 11:**
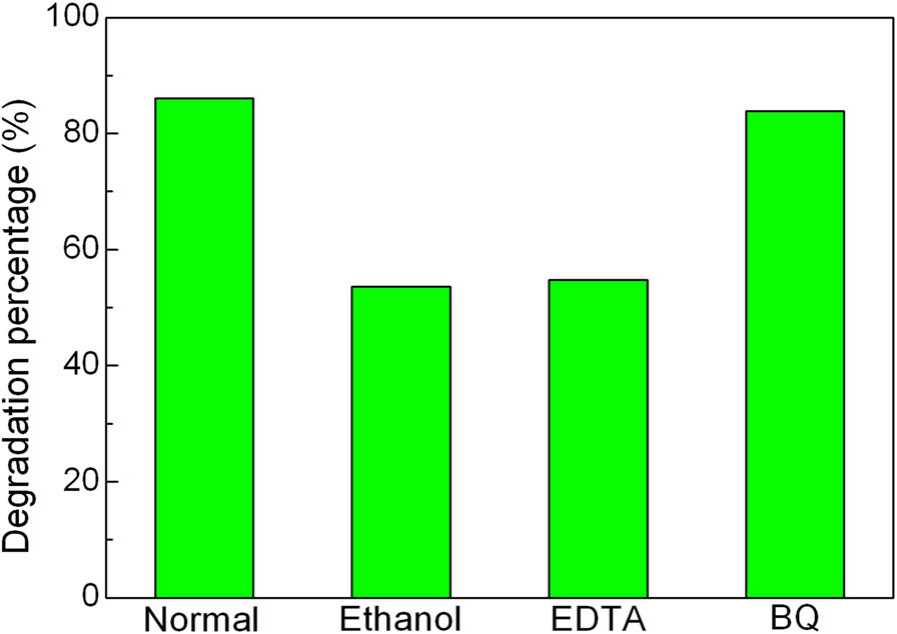
Effects of ethanol, EDTA, and BQ on the degradation percentage of AO7 over 20wt%Ag_3_PO_4_/BiFeO_3_ composite

It is well known that photogenerated hole (h^+^), hydroxyl (•OH), and superoxide (•O^2−^) are considered to the main active species responsible for the photocatalytic degradation of dye. In order to clarify the role of the active species in the present photocatalytic system, the active species trapping experiments were carried out, as shown in Fig. 11. It can be seen that the degradation percentage of AO7 undergoes an obvious decrease after the introduction of ethanol (scavenger of •OH, 10% by volume) or ethylene diamine tetraacetic acid (EDTA, scavenger of h^+^, 2 mM). This indicates that •OH and h^+^ are the major active species involved in the photocatalytic reaction. After the addition of benzoquinone (BQ, scavenger of •O^2−^, 1 mM), a slight decrease of degradation percentage is detected, suggesting that •O^2−^ plays a relatively minor role in the dye degradation.

### Proposed Photocatalytic Mechanism

It is well known that the redox ability and migration of photogenerated charges are highly related to the energy-band potentials of photocatalysts. The valence band (VB) and conduction band (CB) of BiFeO_3_ and Ag_3_PO_4_ can be obtained using the following equation [58, 59]:

4 $$ {E}_{\mathrm{VB}}=X-{E}^{\mathrm{e}}+0.5{E}_{\mathrm{g}} $$

5 $$ {E}_{\mathrm{CB}}=X-{E}^{\mathrm{e}}-0.5{E}_{\mathrm{g}} $$

**Fig. 12:**
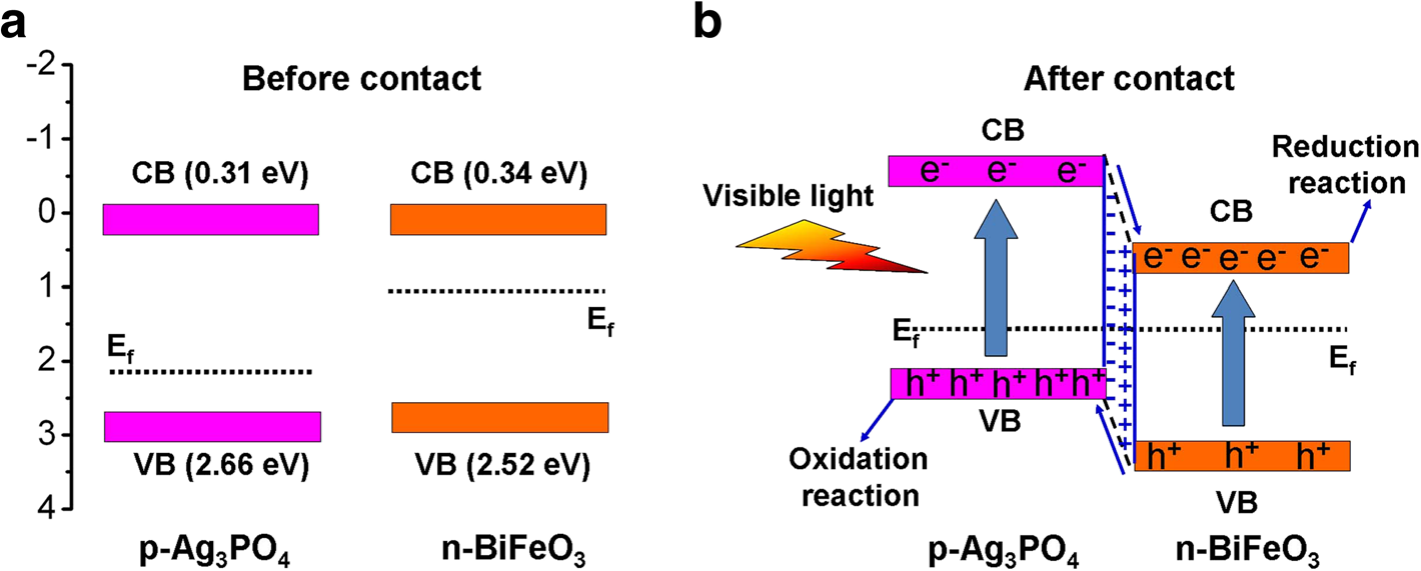
Schematic illustration of proposed photocatalytic mechanism for Ag_3_PO_4_/BiFeO_3_ composite. **a** Before contact. **b** After contact

*X* is the absolute electronegativity of semiconductor (calculated as the arithmetic mean of the electron affinity and the first ionization of the constituent atoms). *E*^e^ is the energy of free electrons on the hydrogen scale (~ 4.5 eV). The *X* values of BiFeO_3_ and Ag_3_PO_4_ are estimated to be 5.93 and 5.98 eV, respectively [43, 60]. Based on Eqs. (4) and (5), the CB/VB potentials of BiFeO_3_ and Ag_3_PO_4_ are calculated to be 0.34/2.52 V and 0.31/2.66 V vs. NHE, respectively. The energy-band potential diagram of the two photocatalysts is shown in Fig. 12a. It is reported that BiFeO_3_ is an n-type semiconductor and its Fermi level lies close to the CB [48]. Ag_3_PO_4_ is demonstrated to be a p-type semiconductor (see Fig. 10c), whose Fermi energy level is close to the VB [43].When BiFeO_3_ is combined with Ag_3_PO_4_ to form p-n heterojunction (see Fig. 12b), the diffusion of electrons and holes between the two photocatalysts will build an internal electric field at the interface region of the p-n heterojunction with direction from BiFeO_3_ to Ag_3_PO_4_. Simultaneously, the energy-band potential of BiFeO_3_ tends to move down along with its Fermi level whereas that of Ag_3_PO_4_ tends to raise up accompanied by its Fermi level until an equilibrium state of Fermi level of the two photocatalysts is achieved. Upon visible-light irradiation, both BiFeO_3_ and Ag_3_PO_4_ can be excited to generate photoinduced electron and hole pairs. Under the promotion of the internal electric field, the photogenerated electrons in the CB of Ag_3_PO_4_ will migrate to the CB of BiFeO_3_, while the photogenerated holes will transfer from the VB of BiFeO_3_ to that of Ag_3_PO_4_. As a result, the recombination of photogenerated charges can be effectively inhibited, as evidenced by the photocurrent and PL analysis (see Fig. 10a, d). Thus, more photogenerated electrons and holes can participate in the photocatalytic redox reaction, leading to the enhancement of the photocatalytic activity for the Ag_3_PO_4_/BiFeO_3_ p-n heterojunction composites.

## Conclusions

Ag_3_PO_4_/BiFeO_3_ p-n heterojunction composites were synthesized through the decoration of Ag_3_PO_4_ spherical-like microparticles on the surface of BiFeO_3_ microcuboids. Compared with bare BiFeO_3_, the as-obtained composites exhibit enhanced visible-light photocatalytic activity for the degradation of AO7 and phenol. Moreover, the composites are demonstrated to be excellent photo-Fenton-like catalysts. The improved photocatalytic activity of the composites is mainly attributed to the efficient separation of photogenerated electrons and holes owing to the formation of the p-n heterojunction between BiFeO_3_ and Ag_3_PO_4._

## Abbreviations

AO7: Acid orange 7
CB: Conduction band
DRS: UV-vis diffuse reflectance spectra
EDX: Energy dispersive X-ray
*E*_g_: Bandgap energy
I-t: Photocurrent-time
NMP: 1-Methyl-2-pyrrolidione
PVDF: Polyvinylidene fluoride
R: Reflectance
SEM: Scanning electron microscope
TEM: Transmission electron microscope
VB: Valence band
XPS: X-ray photoelectron spectroscopy
XRD: X-ray diffractometer
